# Design of Metamaterial Based Efficient Wireless Power Transfer System Utilizing Antenna Topology for Wearable Devices

**DOI:** 10.3390/s21103448

**Published:** 2021-05-15

**Authors:** Tarakeswar Shaw, Gopinath Samanta, Debasis Mitra, Bappaditya Mandal, Robin Augustine

**Affiliations:** 1 Department of Electronics and Telecommunication Engineering, Indian Institute of Engineering Science and Technology, Shibpur, Howrah 711103, West Bengal, India; tarakeswar.shaw@gmail.com (T.S.); debasisiit@gmail.com (D.M.); 2 Department of Electronics and Telecommunication Engineering, C. V. Raman Global University, Bhubaneswar 752054, Odisha, India; gopi.samanta85@gmail.com; 3 Microwaves in Medical Engineering Group, Electrical Engineering, Division of Solid-State Electronics, Uppsala University, 752 36 Uppsala, Sweden; Robin.Augustine@angstrom.uu.se

**Keywords:** wireless power transfer, wearable antenna, electromagnetic radiation, radiative near-field, wearable devices, metamaterial, power transfer efficiency

## Abstract

In this article, the design of an efficient wireless power transfer (WPT) system using antenna-based topology for the applications in wearable devices is presented. To implement the wearable WPT system, a simple circular patch antenna is initially designed on a flexible felt substrate by placing over a three-layer human tissue model to utilize as a receiving element. Meanwhile, a high gain circular patch antenna is also designed in the air environment to use as a transmitter for designing the wearable WPT link. The proposed WPT system is built to operate at the industrial, scientific and medical (ISM) band of 2.40–2.48 GHz. In addition, to improve the power transfer efficiency (PTE) of the system, a metamaterial (MTM) slab built with an array combination of 3 × 3 unit cells has been employed. Further, the performance analysis of the MTM integrated system is performed on the different portions of the human body like hand, head and torso model to present the versatile applicability of the system. Moreover, analysis of the specific absorption rate (SAR) has been performed in different wearable scenarios to show the effect on the human body under the standard recommended limits. Regarding the practical application issues, the performance stability analysis of the proposed system due to the misalignment and flexibility of the Rx antenna is executed. Finally, the prototypes are fabricated and experimental validation is performed on several realistic wearable platforms like three-layer pork tissue slab, human hand, head and body. The simulated and measured result confirms that by using the MTM slab, a significant amount of the PTE improvement is obtained from the proposed system.

## 1. Introduction

In the past few decades, a remarkable advancement in the biomedical field brings a radical change in the health monitoring and treatment of patients. Recently, wearable devices such as body sensors and smart electronic devices are widely used to observe the critical health conditions of patients [1,2,3]. These devices are used to construct a wireless body sensor network (WBSN), which enables constant monitoring and acquisition of physiological data like body temperature, blood pressure, glucose level and electrocardiogram (ECG), etc., of the patients and transfer these vital information’s to the connected healthcare system [4]. However, attaining the practical and functional connectivity between the different sections of the body sensors remains a technical challenge. Usually, wires are broadly used to connect devices in clinical and research settings that disrupt physical activities and not suitable for continuous use. To power the wearable devices, a high demand exists for designing a simple, compact and efficient wireless power transfer (WPT) system that reduced the dependency on battery capacity and eliminates the wire connections around the body.

Since the experimental demonstration of wireless electricity performed by Nikola Tesla in his pioneer work [5], there has been an increasing interest in both industrial and academic communities to design an efficient WPT system [6]. The WPT system finds its resourceful applicability in the field of charging portable devices [7], vehicles [8] and even for implantable medical devices [9] wirelessly. In recent times, significant research efforts have also been given to construct the non-radiative inductive and magnetic resonant coupling-based WPT system [10,11,12,13,14,15,16] for wearable electronic devices. In Refs. [10,11], a magnetic resonance wireless power transfer (MR-WPT) based approach has been presented to power both wearable and implantable devices over a short transfer distance. In these studies [10,11], the transmitting (Tx) and receiving (Rx) elements are designed over the flexible polyester fiber using an adhesive copper sheet. In [12], a non-planar wearable WPT system with improved power transfer through stretchable magnetic composites has been presented. Furthermore, a four coil-based non-planar hand wearable WPT system is designed using magnetic resonance coupling in Ref. [13]. Furthermore, a wearable textile embroidered Rx antenna connected to a rectifier circuit is used to design the MR-WPT system in [14]. Moreover, a comparative study to develop the wearable WPT system on the different dielectric materials is presented using a strongly coupled magnetic resonance approach [15]. However, the use of lumped capacitor and multi-layered (12 layers) Tx structure in [15] increases the design complexity as well as introduces additional losses to the WPT system. Most recently, in [16], a resonant inductive WPT is designed using an embroidered textile coil for a smart cycling glove. The construction of Tx and Rx elements in Refs. [14,16] with perfect accuracy over a textile material using embroidered is quite complicated and cumbersome.

In order to obtain better power transfer efficiency (PTE) from the reported MR-WPT systems [10,11,12,13,14,15,16], the Tx and Rx elements must be excited at the same resonant frequency. Furthermore, the performance of the non-radiative WPT system highly depends upon the magnetic coupling between the Tx and Rx section, and the PTE reduced rapidly due to an increase in transmission distance [17]. In addition, the efficiency of the non-radiative system is highly affected by the lateral and angular misalignment effect between the Tx and Rx elements. These issues of the non-radiative WPT system have limited the practical applicability to power the wearable devices. Hence, an effective technique is highly preferred to power the wearable devices wirelessly by mitigating the overhead issues.

In this regard, the radiating principle of the antenna may also be considered to construct an effective WPT system for wearable devices. This is because; the antenna-based radiative WPT system provides flexibility in transfer distance along with better misalignment tolerance [18]. Though, huge research work has been made by the various groups to construct the radiating principle-based WPT system for air [19] and implantable devices [20,21,22]. However, to the best of our knowledge, any substantial research effort has not been made in the literature to design a radiating principle-based wearable WPT system.

This study aims to address the knowledge gap by designing an antenna topology-based WPT system for wearable devices. To construct the wearable WPT system, two simple circular patch antennas are designed to use as Tx and Rx elements. Further, the PTE of the proposed system has been improved by using a zero-index metamaterial (ZIM) slab consists of both-sided ring unit cells. The performance stability analysis of the proposed system is exhibited on the human hand, head and torso model. The analysis of specific absorption rate (SAR) has been performed to illustrate the effect of radiation on the human body. Furthermore, some metamaterial-based approach has been discussed to control the SAR in the standard limit. Moreover, the performance study of the proposed system due to the misalignment and flexibility of the Rx antenna is accomplished. Finally, to establish the proposed concept, experimental validation is performed with and without MTM slab. The measured and simulated result shows more than 9% efficiency enhancement due to the application of the MTM slab.

## 2. Design of Wearable Wireless Power Transfer System

In this section, to construct the wearable WPT system, the design of the Tx and Rx antenna is presented. Furthermore, the configuration of the simulation setup to construct the WPT link by employing the designed Tx and Rx antenna has been illustrated.

### 2.1. Construction of the Wearable Receiving (Rx) and Transmitting (Tx) Antennas

**Figure 1:**
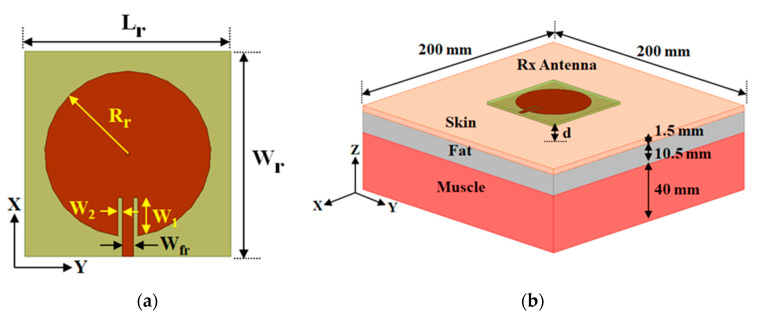
(**a**) Schematic configuration of the Rx patch antenna. (**b**) Three-layer phantom tissue (skin, fat and muscle) model used in HFSS simulator.

In the case of wearable applications, simplicity and better performance are the prime concern for designing the WPT system. Keeping the requirement in mind, initially, we designed a simple inset-fed circular patch antenna over a flexible substrate with a full ground plane to use as an Rx element. The configuration of the Rx patch antenna is shown in Figure 1a. The antenna is constructed over the felt substrate having the permittivity of 1.63 and loss tangent of 0.044 with a thickness of 1 mm. The optimization of the Rx antenna is performed by placing it over the three-layer human tissue model, as shown in Figure 1b. The electrical property of different human tissue is mentioned in Table 1. The dimension of the wearable Rx antenna is adjusted to operate at the ISM of 2.45 GHz. The parameters of the wearable antenna are included in Table 2.

**Figure 2:**
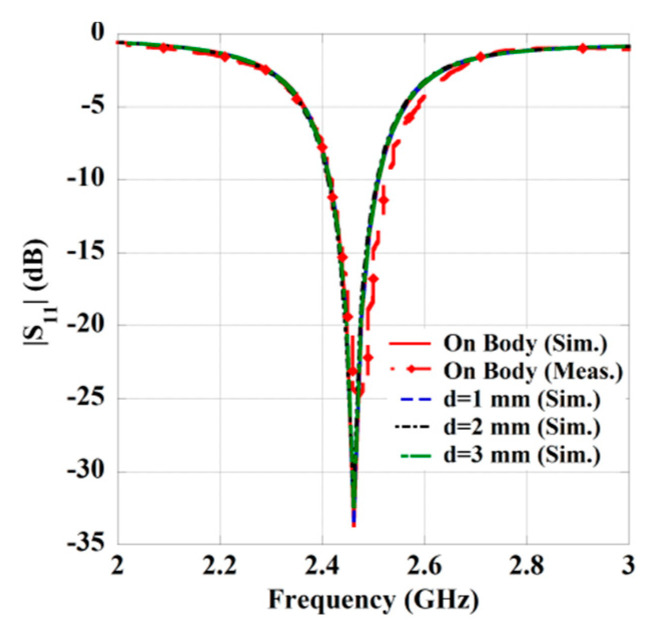
Return loss property of the wearable Rx antenna.

**Figure 3:**
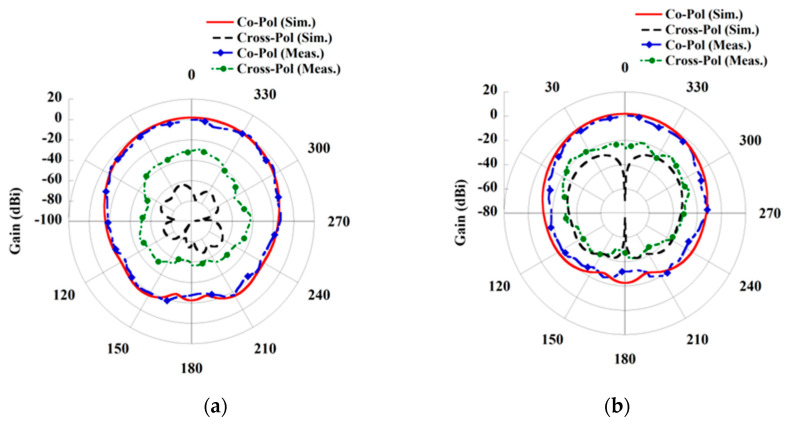
Radiation pattern of Rx antenna. (**a**) E-plane and (**b**) H-plane.

In cases of wearable applications, either the Rx element is directly placed over the human body, or an air gap is considered between the body and antenna. Both states are considered and studied during the design of wearable Rx antenna. The simulated return loss characteristics of the Rx antenna by placing over the body and considering an air gap (*d*) are shown in Figure 2. The measured result by placing the antenna on the three-layered pork slab is also included in Figure 2. A good agreement between the simulated and measured results has been observed. Furthermore, it can be seen from the return loss characteristics that the resonance frequency is retained at 2.45 GHz from simulations and at 2.46 GHz from the measurement. We also investigated the gain of the Rx patch antenna for the on-body and air gap scenario. However, for the sake of clarity, only the simulated and measured E-plane and H-plane radiation pattern of the Rx patch by directly placing on the human skin tissue model (in simulation) and on the pork slab (in measurement) is plotted in Figure 3. The maximum gain attained from the wearable Rx antenna is 1.86 dBi from the simulation, while 0.87 dBi from the measurement. Furthermore, the simulated radiation efficiency of the wearable antenna is 18.37 %, and the full width at half maxima (FWHM) is found to be 70.8°/63.4° in the E/H plane. Moreover, a detailed analysis of the wearable antenna characteristics for both on-body and an air gap (*d*) scenario is presented in Table 3. It can be seen from the tabulated data that although the resonance frequency of the antenna remains the same, but a slight change has been seen in the matching of return loss and gain of the antenna. Such stability obtained from the wearable antenna may be due to the presence of the full ground plane and the broadside directive radiation pattern of the patch. All the simulations are performed by utilizing the commercially available software ANSYS HFSS.

**Figure 4:**
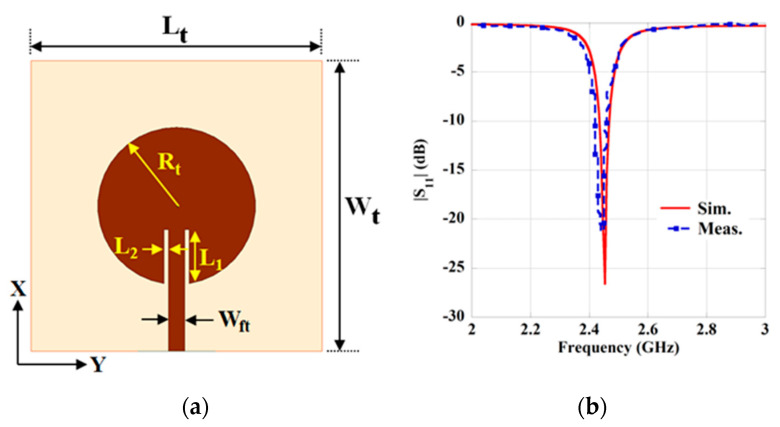
(**a**) Schematic of the Tx antenna. (**b**) Simulated and measured return loss characteristics.

**Figure 5:**
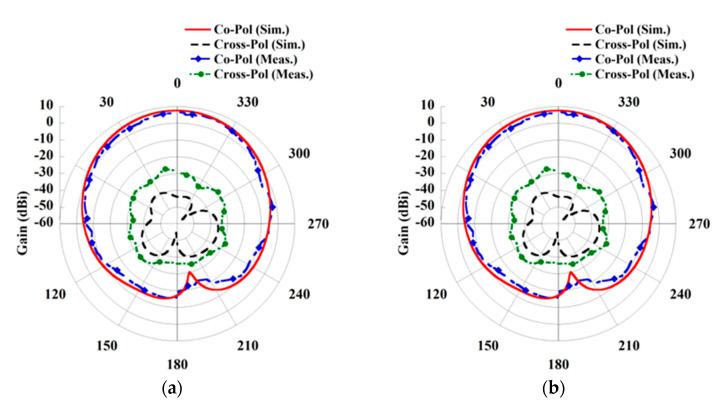
The radiation pattern of the Tx antenna. (**a**) E-plane and (**b**) H-plane.

To construct the WPT link, an inset-fed circular patch antenna with a full ground plane has also been designed to use as a Tx element in the air environment. The geometry of the Tx antenna is shown in Figure 4a. Herein, a patch antenna is utilized as a Tx element due to its simplicity, broadside radiation pattern and capability to integrate with the system easily. To obtain high gain from the Tx antenna, it is designed on a low-loss dielectric substrate having the dielectric permittivity of 2.2 and loss tangent 0.0009 with a thickness of 1.58 mm. The dimension of the Tx antenna is also optimized to operate at the same frequency (2.45 GHz) of the Rx element to obtain better performance from the designed system. The optimized parameter of the Tx antenna to operate at 2.45 GHz is given in Table 4. Furthermore, the simulated and measured return loss property of the Tx patch is shown in Figure 4b. The resonance frequency obtained from the measured result is 2.44 GHz for the antenna. Further, the simulated and measured radiation pattern characteristic is illustrated in Figure 5. The maximum gain achieved from the Tx antenna is 7.56 dBi from the simulation, while 7.13 dBi is achieved from the measurement. Moreover, the simulated radiation efficiency of the Tx antenna is 92.7%, and the FWHM is found to be 74.7°/75.1° in the E/H plane.

### 2.2. Design of Wearable WPT Link

**Figure 6:**
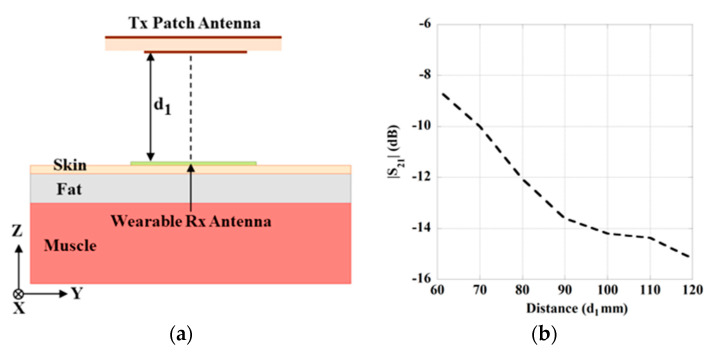
(**a**) Schematic of the wearable WPT system. (**b**) Simulated result for the variation of transmission strength (|S_21_|) with transfer distance (*d*_1_).

The schematic configuration of the WPT system by integrating the designed Tx and Rx antennas from the previous section is presented in Figure 6a. The distance between the Tx antenna and the surface of Rx antenna is marked by *d*_1_. The transfer distance (*d*_1_) has been chosen for the proposed system to operate in the radiative near-field region of the Tx antenna. In this region, the radiated beams are confined that provides better system performance with enhanced transmission distance and less sensitive to misalignments [19]. The variation of transmission characteristics (|*S*_21_|) with the increase of transmission distance (*d*_1_) in the near-field region is presented in Figure 6b. It can be observed from the figure that the strength of |*S*_21_| is decreased with the increase of *d*_1_. This is due to the spreading nature of radiating waves and unwanted attenuation in the free space of the Tx antenna. It can also be seen from the figure that 60 mm distance provides the high value of |S_21_| (–8.54 dB), which offers better PTE. In the next section, a metamaterial slab is designed and utilized to concentrate or reduce the spreading nature of the radiating waves from the Tx antenna to improve the PTE of the system.

## 3. Metamaterial Integrated Proposed Wearable WPT System

**Figure 7:**
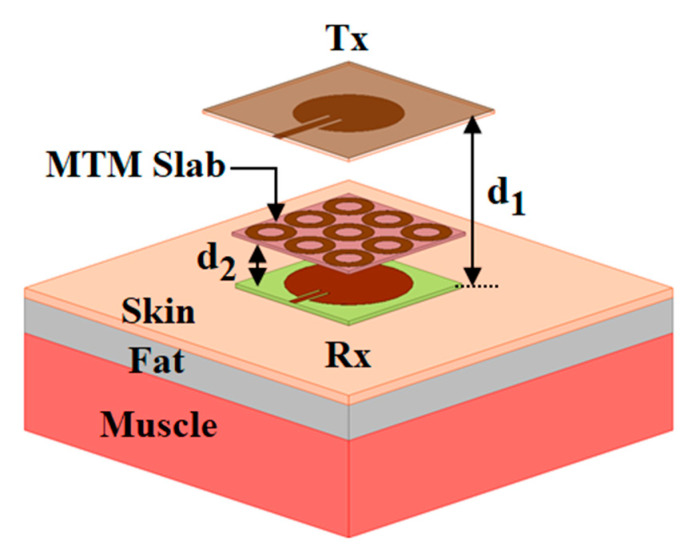
Configuration of the proposed MTM-based wearable WPT system.

**Figure 8:**
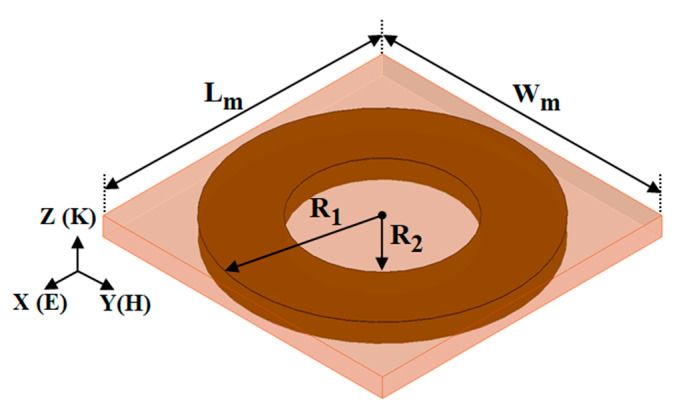
Schematic view of the both-sided ring structure. Where L_M_ = W_M_ = 24 mm, R_1_ = 11.25 mm and R_2_ = 6 mm.

**Figure 9:**
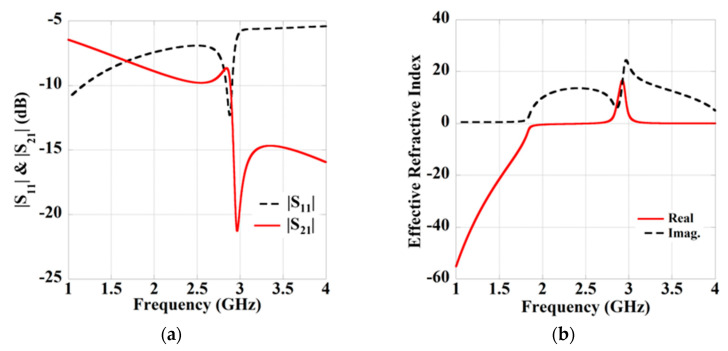
(**a**) Property of the transmission and reflection coefficient of the ring unit cell. (**b**) Extracted effective refractive index.

The proposed wearable WPT systems integrated with an MTM slab containing an array of 3 × 3 both-sided ring unit cell is shown in Figure 7. The MTM is placed at a distance of *d*_2_ from the wearable Rx antenna. The both-sided MTM ring unit cell configuration is used to improve the performance of the proposed WPT system is shown in Figure 8. In the figure, the orientation of the exciting electromagnetic wave to the MTM structure is also presented. The MTM structure is designed on an inexpensive FR4 dielectric substrate, having the permittivity of 4.4, loss tangent of 0.02 with a thickness of 1.6 mm. The dimensions of the MTM structure are optimized to obtain zero-index property at 2.45 GHz by following the methodology discussed in [25]. The optimized parameters of the ring structure are included in the caption of Figure 8. The transmission (|S_21_|) and reflection (|S_11_|) characteristic of the ring unit cell is shown in Figure 9a. The extracted effective refractive index property of the unit cell is also shown in Figure 9b. The parameter extraction is performed by the approach mentioned in [26]. It can be observed from Figure 9b that the ring unit cell provides the zero-index characteristic over the entire ISM (2.40–2.48 GHz) frequency band. Herein, the efficiency of the proposed system is improved by concentrating the radiating energy of the Tx antenna over the Rx antenna utilizing the zero-index behaviour of the MTM slab. As per Snell’s law, when a spreading electromagnetic wave passes through a zero-index slab, the refracted wave becomes normal to the slab. Henceforth by utilizing the MTM slab, the gain of the Tx antenna is enhanced by focusing the incoming wave in the direction of broadside. Subsequently, the efficiency of the wearable WPT is enhanced significantly.

The performance enhancement of the WPT system highly depends on the combination of the unit cell array and its placement position (*d*_2_) from the receiving antenna [21,22]. Henceforth, a parametric study has been performed to find out the optimal combination of unit cell array as well as its position, *d*_2_, that provides the better performance enhancement from the system. The simulated result for the various combinations of the unit cell array is presented in Table 5. From the table, it can be seen that the combination of 3 × 3 arrays offers a better increment (2.88 dB) in coupling strength (|∆S_21_|) that enhances the PTE of the system noticeably compared to other combinations. The optimal performance enhancement is obtained for the array combination of 3 × 3 due to the uniform illumination effect of the MTM slab discussed in [27]. It can also be realized from Table 5 that due to increment in array combination, the performance of the system is degraded. This is because a single Tx patch antenna cannot effectively illuminate the large panel size of the MTM slab, which generates a loss in the system [21,27]. Furthermore, the effect of the MTM-slab (with 3 × 3 arrays) for the different placement positions (*d*_2_) in the proposed WPT system is presented in Table 6. The optimization of *d*_2_ is performed to diminish the near field coupling effect between the MTM slab and Tx patch. It can be perceived from the table that at 8 mm distance, better enhancement in coupling strength (|∆S_21_|) is attained. The improvement in coupling is attributed to better impedance matching among the MTM slab and the Tx patch at the specified distance [21,22,27].

**Figure 10:**
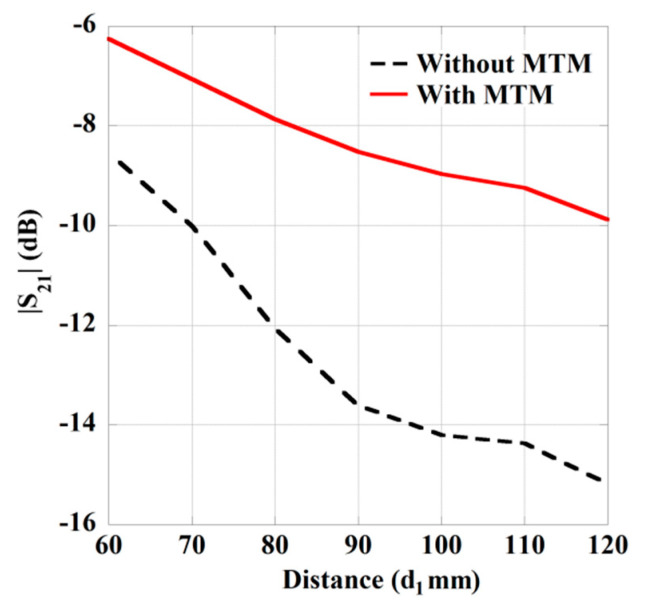
Variation in the transmission strength with and without MTM slab.

**Figure 11:**
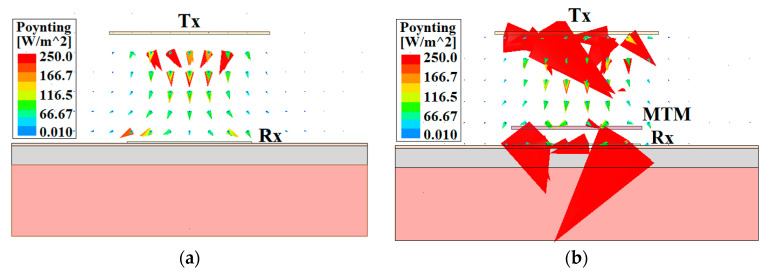
Distribution of poynting vector. (**a**) Without and (**b**) with MTM slab.

Furthermore, to demonstrate the effect of the zero-index MTM slab on the transmission strength (|*S*_21_|) of the WPT system, a comparative study is performed with and without the integration of the MTM slab. The comparison of |S_21_|-characteristic is illustrated in Figure 10, with the variation of transfer distance (*d*_1_). It can be clearly perceived from the figure that the transmission strength is enhanced significantly along with the change of transmission distance due to the use of MTM slab. Consequently, the efficiency of the proposed wearable system is enhanced considerably. Furthermore, to clarify the reason behind the performance improvement of the proposed MTM integrated WPT system, the Poynting vector distribution for without and with MTM integrated system is depicted in Figure 11a,b, respectively. From the figure, it can be seen that due to the integration of MTM slab, the radiated electromagnetic wave from the Tx antenna is concentrated over the wearable Rx antenna with higher intensity, which improves the performance of the system considerably.

**Figure 12:**
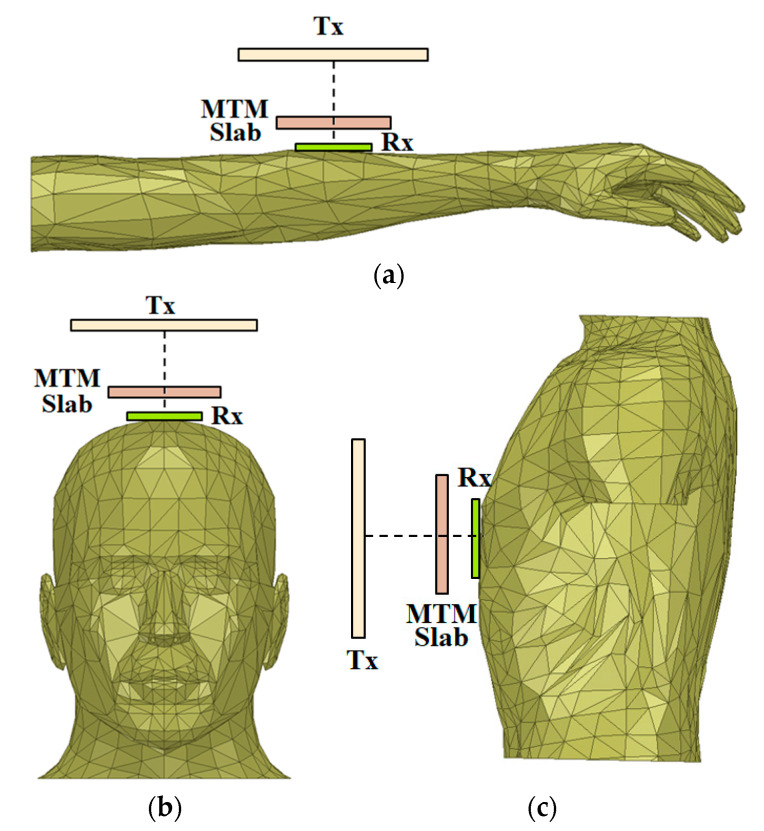
The scenario of the proposed MTM integrated wearable WPT system over human, (**a**) hand, (**b**) head and (**c**) torso model.

**Figure 13:**
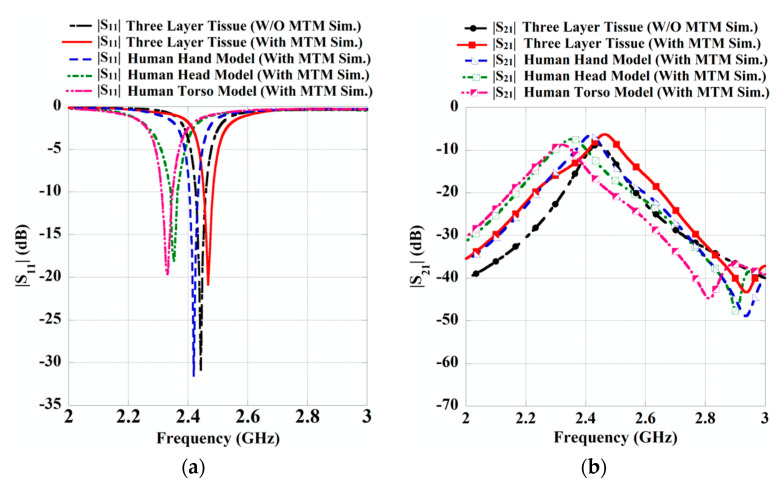
Comparison in S-parameters for various wearable scenarios such as three-layer tissue, human hand, head and torso model. (**a**) Reflection and (**b**) transmission characteristics.

Moreover, a comparative performance analysis of the MTM integrated wearable WPT system is done by positioning the Rx antenna over the voxel models like human hand, head and torso. The simulation setup by placing the Rx antenna on the different portions of the human body is shown in Figure 12. This study is accomplished with the optimized 3 × 3 array combination of the unit cell on the MTM slab at *d*_1_ = 60 mm and *d*_2_ = 8 mm. Figure 13a,b depicts the S-parameter properties of the proposed wearable WPT system applied to different models. From Figure 13b, it can be observed that the strength of the transmission coefficient (|S_21_|) is enhanced significantly due to the use of MTM slab, which improves the efficiency of the system. For the sake of clarity, the S-parameter plots for the without MTM slab in case of the human hand, head and torso model are not incorporated in Figure 13. However, a detailed comparative study for the different wearable scenarios with and without the integration of the MTM slab is furnished in Table 7. Herein, the efficiency of the WPT is calculated from the transmission characteristics (|S_21_|) as, *η* = |S_21_|^2^ [20,21,22]. A change in efficiency improvement, along with a shift in operating frequency, is observed from Table 7. Such variation occurs as the human body is a most complex environment with the discrepant dielectric properties of various parts of human tissues [28], which usually affects the system’s performance differently. In the proposed wearable WPT system, the performance is analyzed for a homogeneous three-layer tissue model as well as for inhomogeneous and irregular structures such as the human hand, head and torso model. Even though the excitation power is kept the same for the Tx antenna, but the dielectric property, size and geometry of the different wearable scenarios vary simultaneously. Henceforth, a variation in the transmission efficiency improvements and shift in operating frequency is observed in Table 7. From the comparative study for the different wearable scenarios, it is clear that the proposed WPT system may be considered for application in the various wearable applications.

## 4. Misalignment and Bending Analysis of the Proposed MTM Integrated System

In the application scenario, it is necessary to estimate the performance stability of the proposed WPT system due to the movement of the human body. Hence, the stability of the system performance is investigated in light of the misalignment between the Tx and Rx antenna. Furthermore, the flexibility effect of the wearable Rx antenna on the system performance is studied.

### 4.1. Misalignment Analysis

**Figure 14:**
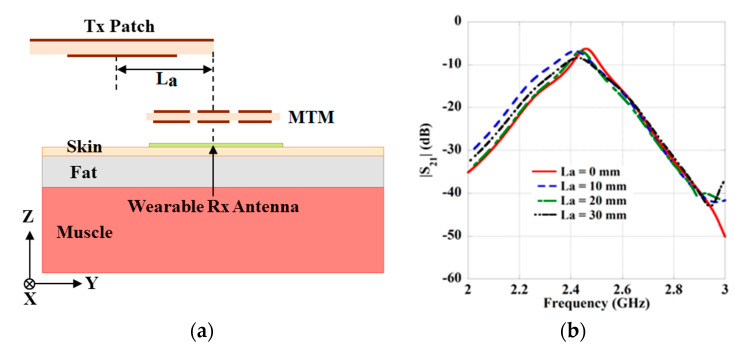
(**a**) Configuration of lateral misalignment of the Tx antenna. (**b**) Effect on transmission strength (|S_21_|).

Perfect alignment among the Tx and Rx antenna is difficult due to the movement of the human posture. Henceforth, the lateral and angular misalignment analysis of the Tx antenna is performed to show the effect on the performance of the proposed WPT system. The analysis of the lateral misalignment is performed by moving the Tx antenna parallelly by keeping the MTM-slab and Rx antenna fixed at their positions, as shown in Figure 14a. In the figure, the lateral offset distance of the Tx antenna is specified by ‘L_a_’. A comparative study about the coupling strengths (|S_21_|) between the Tx and Rx antenna with the variation of L_a_ is presented in Figure 14b. Furthermore, in Table 8, the effect of lateral alignment on the operating frequency and transmission strength is clearly presented. It can be perceived from the table that with the enhancement of the ‘L_a_’, the transmission strength of the system is degraded along with a frequency shift.

**Figure 15:**
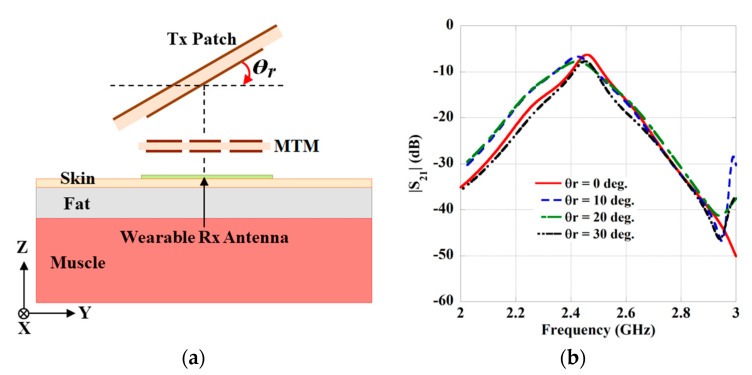
(**a**) Schematic of the angular misalignment of the Tx antenna. (**b**) Effect on transmission strength (|S_21_|).

Furthermore, the study of angular misalignment is also executed by rotating the Tx antenna in the yz-plane by keeping the MTM-slab and Rx antenna fixed at their positions, as represented in Figure 15a. The change in transmission strength (|S_21_|) is also studied for the different values of rotation angle (*θ*_r_) and presented in Figure 15b. Moreover, for a better understanding about the effect of angular misalignment, a comparative study is given in Table 9. A slight change in the coupling strengths (|S_21_|) can be observed from the table.

### 4.2. Bending Analysis

**Figure 16:**
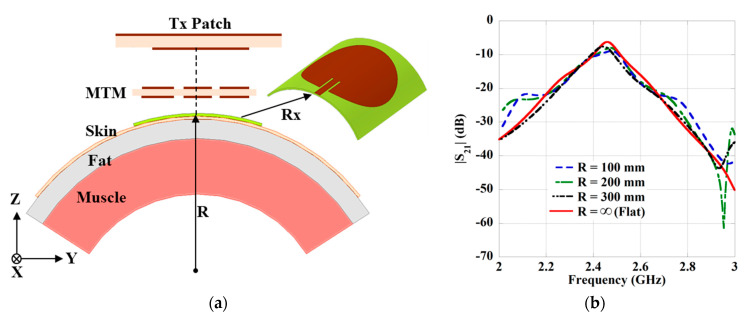
(**a**) Geometry of the bending study of the Rx antenna. (**b**) Effect on transmission (|S_21_|) strength.

The wearable Rx antenna has been designed over a flexible felt substrate to eliminate the difficulty faced with the rigid substrate during the integration on the human body. To analyze the flexibility effect, the Rx antenna has been wrapped around the three-layer cylindrical tissue model of radius R, illustrated in Figure 16a. The variation in flexibility of the Rx antenna is performed by changing the radius R. The effect of flexibility on the coupling strength (|S_21_|) is shown in Figure 16b, with the variation of R. From the figure, a minor change can be perceived due to the flexibility of the Rx antenna.

## 5. Analysis of Specific Absorption Rate (SAR)

The safety of the human body and health is the prime concern against the harmful effects of the electromagnetic (EM) field for human exposure. Therefore, the major challenge regarding the design of an efficient WPT system, particularly for the wearable WPT system, is to maintain the safety standards. Regarding safety, the value of SAR would be taken into consideration for the transmission of high power at enhanced transmission distances. Generally, the SAR is defined by the rate of absorbed energy within the unit mass of the human body when explored in the EM field. The SAR value is also expressed mathematically by using the following equation [29],

(1) $$\mathrm{SAR}\;=\frac{\sigma {\left|E\right|}^{2}}{\rho }\left[W/\mathrm{kg}\right]$$

where σ is the tissue conductivity (S/m), *E* is the electric field density (V/m), and ρ is the intensity of tissue (kg/m^3^). It can be realized from the above expression that the SAR value depends upon the property of human tissue and its density along with the induced E-field from the Tx element. Furthermore, the level of EM radiation absorbed by the human body is directly related to the conductivity of the human organs. Usually, the tissue of human body consists with a lot of water that increases the conductivity. Henceforth, the SAR varies on the different body parts of the human [30].

**Figure 17:**
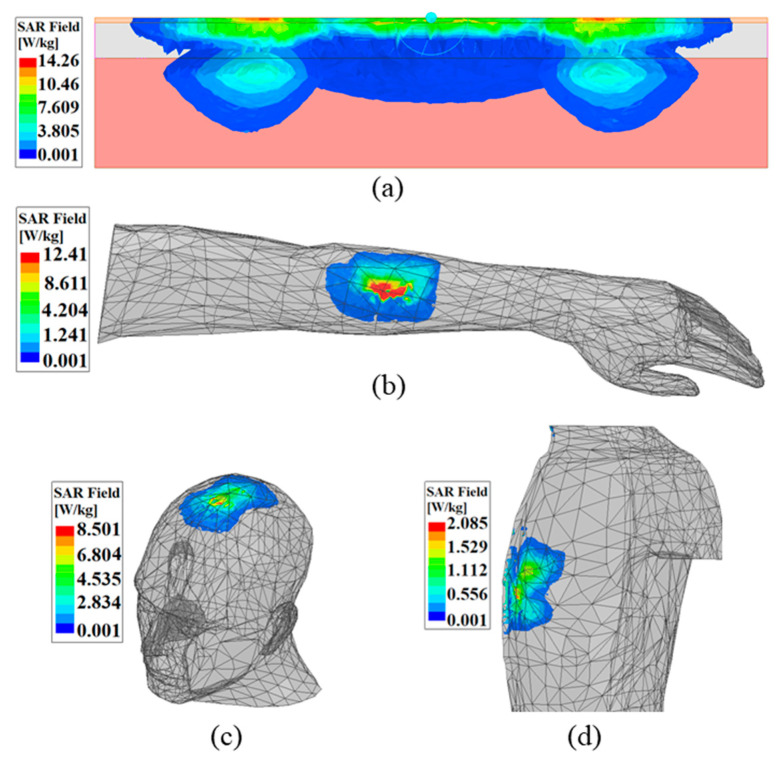
Distribution of SAR for different wearable applications. (**a**) Three-layer tissue, (**b**) human hand, (**c**) head and (**d**) torso model.

In the present study, the 1-g average SAR distribution for the proposed MTM integrated WPT system for the different wearable scenarios is illustrated in Figure 17. From the figure, the obtained peak SAR value in the case of the three-layer tissue, hand, head and torso model are 14.26, 12.41, 8.50 and 2.08 W/kg, respectively. However, regarding the safety of the human body, the maximum average SAR value over the 1-g of cubic tissue model allowed by the IEEE C95.1−1999 standard should be less than or equal to 1.6 W/kg [31]. As per the recommendation of the Federal Communications Commission’s (FCC) standards [32], the maximum allowed transmitter output energy fed to the Rx antenna at the ISM-bands is at 1 W (30 dBm). Herein, the distribution of the SAR is performed for the maximum 1 W excitation power set at the Tx patch. Moreover, by decreasing the excitation power at the transmitter end, the peak 1-g average SAR value should be kept within the standard limit [20,21,22]. The excitation power required at the Tx end to keep the SAR value in safety limits in the case of the three-layer tissue, hand, head and torso model are 0.11, 0.13, 0.19 and 0.78 W, respectively.

Furthermore, the novel property of metamaterial has also been utilized to reduce the SAR [33,34,35,36] without reducing the excitation power at the Tx end. In [33], an MTM structure consists of split-ring resonators (SRRs) has been used to reduce the electromagnetic interface between the antenna and the human head. Furthermore, an MTM-based perfect artificial magnetic conductor (AMC) has been used to minimize the SAR of a planar inverted-F antenna [34]. Further, the performance enhancement and SAR reduction of a dual-band flexible antenna have been achieved in [35] by using an MTM-slab at the backside of antenna in WBAN application. In [36], an MTM reflector was recently designed on a felt textile substrate to reduce the SAR effect and increase the performance of an ultra-wideband antenna for WBAN applications. Moreover, the SAR value of the proposed MTM based wearable WPT system might also be reduced by the approach presented in Refs. [33,34,35,36].

## 6. Measurement and Discussion

**Figure 18:**
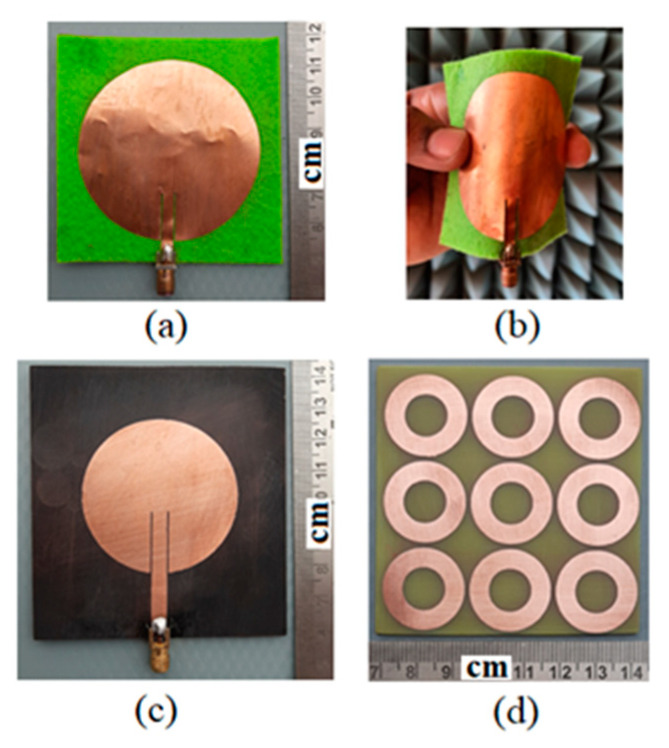
Photographs of the fabricated prototypes. (**a**) Receiving wearable patch antenna, (**b**) flexibility of the wearable antenna, (**c**) transmitting patch and (**d**) MTM slab with 3 × 3 array of unit cells.

**Figure 19:**
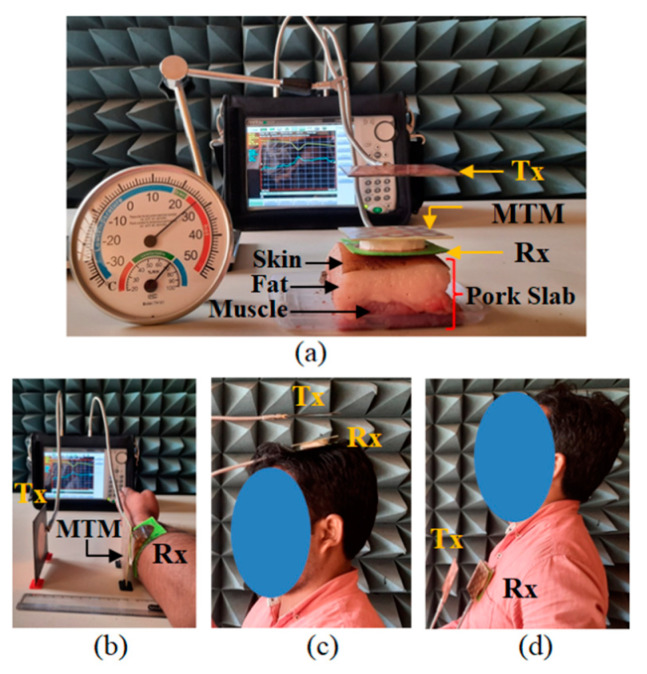
Measurement in different environments. (**a**) Three-layer pork slab, (**b**) human hand, (**c**) head and (**d**) body (chest).

To validate the proposed design concept of the wearable WPT system, the different parts such as Tx, Rx antenna, and the array of 3 × 3 MTM slab have been fabricated. The standard printed circuit board (PCB) technology has been used to design the Tx antenna and MTM slab. The wearable Rx antenna is designed over a felt substrate using an adhesive copper sheet with a thickness of 0.035 mm. To sustain the conductivity of uniform surface, a single copper sheet has been utilized to construct both the full ground and radiating patch along with microstrip feed without any discontinuity or interconnects. The photographs of the fabricated prototype to build the wearable WPT link are shown in Figure 18. The three-layer pork tissue has been used to consider the realistic multi-layered environment for measurement purpose [23]. Furthermore, the measurements are conducted by placing the wearable antenna over the human hand, head and body (chest). The pictures of measurements regarding the realistic scenario of the wearable platforms like the three-layered pork slab and also at the different portions of the human body integrated with the MTM slab are presented in Figure 19. The MTM slab has been attached over the wearable Rx patch antenna by using Styrofoam. The measurements are performed with the help of the Anritsu S820E vector network analyzer.

**Figure 20:**
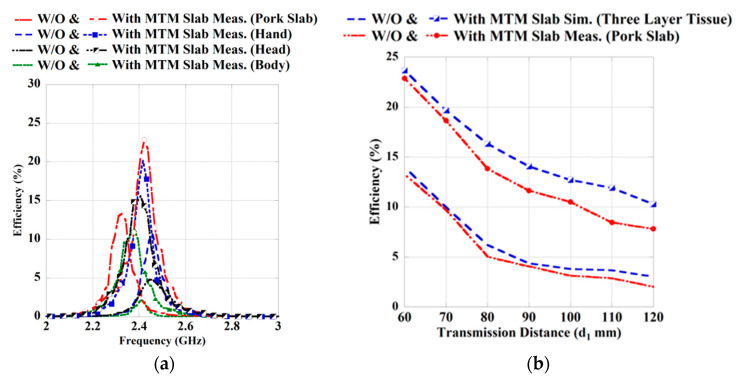
Measured and simulated PTE characteristics in the different wearable scenarios with and without MTM slab, (**a**) at *d*_1_ = 60 mm and (**b**) at different transfer distances.

During measurement, the distance (*d*_1_) between Tx and Rx antenna is considered 60 mm, whereas the MTM slab is placed at a distance (*d*_2_) of 8 mm from the wearable Rx antenna, as shown in Figure 19. The Tx, Rx antenna and MTM slab are aligned properly on the same horizontal line during the experiment to obtain better performance from the system. A comparative study between the measured efficiency in the different scenarios for without (W/O) and with the integration of MTM slab is shown in Figure 20a. It can be found from the figure that for the use of MTM slab, the efficiency of the proposed system is improved significantly. Herein, the efficiency has been calculated from the measured transmission (|S_21_|) coefficient characteristics. Furthermore, the detailed analysis of the obtained measured results from the different wearable scenarios with and without the integration of the MTM slab is summarized in Table 10. From the table, it can be observed that the measured coupling strength |*S*_21_| is improved significantly with the presence of MTM slab, which enhances the efficiency of the system. The improvements in the measured efficiencies are 9.62%, 9.61%, 10.6% and 8.2% in the case of three-layer pork slab, human hand, head and body (chest), respectively.

Furthermore, to present the MTM effect on the PTE of the system, transfer distance (*d*_1_) is increased, keeping the value of *d_2_* fixed at 8 mm in the measurement scenario of the three-layer pork slab. The simulated and measured efficiency plot of the WPT system for the three-layer phantom model and pork slab environment is also presented in Figure 20b. From the figure, it can be observed that by using the ZIM slab, the efficiency level of the proposed WPT system is improved significantly, even with the increase in transmission distance. However, some extent of mismatch is seen between the measured and simulated results, which occurred due to the fabrication tolerance as well as misalignment during measurement.

Finally, a comparative study has been furnished in Table 11 with the proposed work and works reported in the literature. From the table, it can be perceived that the reported work in the literature usually employs the non-radiative inductive or resonant coupling-based approach to design the wearable WPT system, whereas, in the present study, antenna topology has been adopted. The proposed system has been constructed on a compact, flexible substrate using a simple design configuration. The system is also applicable for different wearable scenarios such as the human hand, head and body.

## 7. Conclusions

This article introduces an efficient approach to construct a WPT system employing the radiating characteristics of the antenna for the applications of wearable devices. In the proposed system, the simplest configuration of the Tx and Rx antennas are employed to construct the wearable WPT link, which would be suitable for practical applications. The system is designed to operate in the radiative near-field region of the antennas that provide flexibility in the transmission distance. An MTM slab having the zero-index property is successfully utilized to improve the PTE of the system. Herein. for the first time, MTM structure has been employed to improve the efficiency of the antenna topology-based radiative WPT system, particularly to charge the wearable devices. Furthermore, stable performance is reached from the proposed WPT system irrespective to the misalignments and bending effect of the Rx antenna up to a limit. Further, considering the safety of the human body from EM explore, the study of the SAR is performed in detail for the different wearable environments. Moreover, the analysis of simulated and measured results confirms the feasibility of the proposed concept to improve the PTE of the WPT system. It is highly expected that the presented radiation-based WPT approach to charge wearable devices brings exciting possibilities in the near future.

## Figures and Tables

**Table 1:** Electric property of human tissue model at 2.45 GHz [23,24].

Tissue Model (Thickness)	Relative Permittivity (ε_r_)	Conductivity, σ (S/m)
Skin (1.5 mm)	38.0	1.46
Fat (10.5 mm)	5.28	0.104
Muscle (40 mm)	52.73	1.74

**Table 2:** Dimensions of the Rx wearable patch antenna.

L_r_ = W_r_	R_r_	W_1_	W_2_	W_fr_
70 mm	28.2 mm	13 mm	0.5 mm	3.7 mm

**Table 3:** Simulated performance of the Rx antenna due to placement on the body and considering an air gap (*d*).

*d* (mm)	Operating Frequency (GHz)	|S_11_| (dB)	Gain (dBi)
0 (On Body)	2.45	−33.71	1.86
1	2.45	−33.16	1.87
2	2.45	−32.81	1.89
3	2.45	−31.60	1.92

**Table 4:** Dimensions of the Tx patch antenna.

You	R_t_	L_1_	L_2_	W_ft_
90 mm	24.4 mm	19.3 mm	0.3 mm	4.88 mm

**Table 5:** Simulated results for the different combination of unit cell arrays on MTM-slab (|S_21_|= −8.54 dB without MTM at *d*_1_ = 60 mm).

Array Combination	Frequency (GHz)	|S_11_| (dB)	|S_21_| (dB)	Increment |∆S_21_| (dB)
3 × 3	2.46	−20.86	−6.26	2.88
4 × 4	2.45	−16.03	−8.16	0.38
6 × 6	2.44	−14.47	−8.35	0.19

**Table 6:** Simulated results for the different placement position (*d*_2_) of the MTM-slab (|S_21_|= −8.54 dB without MTM at *d*_1_ = 60 mm).

*d*_2_ (mm)	Frequency (GHz)	|S_11_| (dB)	|S_21_| (dB)	Increment |∆S_21_| (dB)
6	2.43	−17.01	−6.36	2.18
8	2.46	−20.86	−6.26	2.88
10	2.42	−16.47	−6.98	1.56

**Table 7:** Comparative study of the performance for the different wearable scenario with and without (W/O) the integration of the MTM slab.

Wearable Environment	MTM Slab Loading	Operating Frequency (GHz)	|S_11_| (dB)	|S_21_| (dB)	Efficiency (%)	Efficiency Improvement (%)
Three-layer Tissue Model	W/O MTM	2.44	−31.12	−8.54	13.94	-
	With MTM	2.46	−20.86	−6.26	23.66	9.72
Human Hand	W/O MTM	2.41	−17.16	−9.10	12.30	-
	With MTM	2.42	−31.54	−6.51	22.34	11.04
Human Head	W/O MTM	2.32	−18.24	−12.64	5.45	-
	With MTM	2.36	−19.17	−7.44	18.03	12.58
Human Torso	W/O MTM	2.30	−22.19	−14.36	3.67	-
	With MTM	2.33	−19.36	−8.74	13.37	9.7

**Table 8:** Effect of the lateral misalignment (L_a_) on the performance of the proposed WPT system.

L_a_ (mm)	0	10	20	30
Frequency (GHz)	2.46	2.41	2.44	2.43
|S_21_| (dB)	−6.26	−6.85	−6.89	−7.38

**Table 9:** Effect of the angular misalignment (*θ*_r_) on the performance of the proposed WPT system.

*θ*_r_ (degree)	0	10	20	30
Frequency (GHz)	2.46	2.43	2.42	2.45
|S_21_| (dB)	−6.26	−6.69	−7.79	−7.89

**Table 10:** Comparative study between the measured results for the different wearable environment with and without MTM slab.

Measured Environment	MTM Slab Loading	Operating Frequency (GHz)	|S_11_| (dB)	|S_21_| (dB)	Efficiency (%)	Efficiency Improvement (%)
Pork Slab	W/O MTM	2.47	−22.65	−8.78	13.24	-
	With MTM	2.42	−17.20	−6.41	22.86	9.62
Hand	W/O MTM	2.45	−16.82	−9.87	10.30	-
	With MTM	2.41	−25.487	−7.01	19.91	9.61
Head	W/O MTM	2.44	−18.36	−13.20	4.78	-
	With MTM	2.40	−23.24	−8.13	15.38	10.60
Body (Chest)	W/O MTM	2.41	−15.28	−15.12	3.07	-
	With MTM	2.38	−18.70	−9.48	11.27	8.20

**Table 11:** Comparative study between the proposed work and works reported in the literature.

Ref.	Size of the Rx (mm × mm × mm)	Operating Frequency	Flexibility of Rx	Design Complexity of Rx	Transfer Distance (mm)	Efficiency (%)	Application Scenario
[10]	90 × 90 × 1.5	6.78 MHz	Yes	Low	60	29.4	Muscle model
[13]	$\pi \times 14^{2}\times 4$	14.5 MHz	No	High	20	45	Arm model
[14]	120 × 80 × 0.787	6.78 MHz	Yes	High	150	46.2	Human body model
[15]	$\pi \times 32^{2}\times 0.75$	80 MHz	Both flexible and rigid	Moderate	60	50	Human hand model
[16]	60 × 60 × 0.266	6.78 MHz	Yes	High	10	64	Human hand model
This work	70 × 70 × 1	2.46 GHz	Yes	Low	60	23.66 (Sim.) 22.86 (Meas.) Pork slab	Human hand, head and body

## Data Availability

Not applicable.
